# Use of over the counter products in older cardiovascular patients admitted to a tertiary care center in USA

**DOI:** 10.1186/s12877-018-0989-7

**Published:** 2018-12-04

**Authors:** Marwan Sheikh-Taha, Hani Dimassi

**Affiliations:** 1 Department of Pharmacy Practice, Lebanese American University, Byblos, Lebanon; 2 Department of Pharmaceutical Sciences, Lebanese American University, Byblos, Lebanon

**Keywords:** Over the counter medications, Cardiovascular disease, Geriatrics

## Abstract

**Background:**

In recent years there has been a substantial increase in the use of over-the-counter (OTC) products around the world. While they are assumed to be safe by consumers, they can potentially lead to adverse effects and drug interactions particularly in older adults.

**Methods:**

We assessed the patterns of OTC products used by older adults admitted to the cardiology service in a tertiary care medical center in the USA over a three month period. We conducted a retrospective chart review where older adults with cardiovascular diseases (CVD) who were taking at least one OTC product at home were included.

**Results:**

Out of 404 patients who were admitted to the cardiology service, 281 (69.6%) were taking OTC products. Patients were taking a total of 659 OTC products; mean of 2.35 ± 1.57 and the range varied from 1 to 9 products. The most commonly used products were vitamins (37.3%), followed by laxatives (17%), minerals (13.6%), stomach acid reducers (9%), and analgesics (3.6%). OTC users were found to be suffering from more comorbidities and received more prescription medications as compared to non-users. Gender and age did not have an impact on the use of OTC products while patients with atrial fibrillation, sleep apnea and gastro-esophageal reflux disease were more likely to use OTC products.

**Conclusion:**

Use of OTC products is quite frequent in older adults with CVD in our study. Clinicians should ask about OTC product usage and counsel patients about the risks and benefits associated with their use.

## Background

The American Heart Association estimates that 92.1 million US adults have at least 1 type of cardiovascular disease (CVD) and by 2030, 43.9% of the US population is projected to have some form of CVD [1]. CVD imposes an enormous burden in terms of disability, mortality, morbidity, functional decline, and healthcare costs specifically in older adults as there is a marked increase in the incidence and prevalence of CVD in this age group [1]. In recent years, there has been an increasing prevalence of using over-the-counter (OTC) products, including nonprescription medications, vitamins, minerals, and herbal products [2, 3]. These products can be purchased without a prescription for therapeutic benefit and preventive healthcare purposes. While OTC products are assumed to be safe by consumers, they can potentially lead to adverse effects and drug interactions particularly in the older adults due to polypharmacy and the changes in renal and hepatic function associated with older age. Furthermore, herbal use is independently associated with low medication adherence among patients with CVD [4].

In the United States, a direct mail out questionnaire showed the prevalence of multivitamin use to be 68% among outpatient with CVD [5]. Additionally, in elderly patients with heart failure, routine use of OTC medications was reported to be 93.3% while herbal therapy use was 11.5% [6]. In Canada, the use of OTC drugs in patients with CVD was reported to be 67% [7]. In Italy, OTC drug use (excluding vitamins and nutritional supplements) among heart failure patients was 75.8% [8].

While the number of products that can be purchased from pharmacies without the need for a prescription is increasing, this may encourage patients to utilize more of these products which are indeed a therapeutic option, but only if used properly under the guidance of a healthcare professional. Unfortunately, physicians are frequently unaware of their patients’ OTC product use because they do not ask patients, and/or patients do not disclose use of OTC products [9, 10].

Literature regarding OTC products use in patients with CVD is limited. Hence, we conducted this study to describe the pattern of use of OTC products in older adults with CVD admitted to a large tertiary care hospital. We also sought to identify variables associated with their use.

## Methods

A retrospective chart review was conducted where all patients with CVD (including coronary heart disease, peripheral arterial disease, heart failure, valvular heart disease, arrhythmia, and stroke) admitted to the cardiology service at a tertiary care center, Huntsville Hospital, Alabama, USA, from March to May 2016 were assessed. Inclusion criteria were patient age of 65 years and above, taking at least one OTC product at home (including nonprescription medications, vitamins, minerals, and herbal products), history of CVD, and admission to the cardiology service. If a patient was admitted more than once during the study period, the first admission was considered the index visit. Data collected from medical records were analyzed using SPSS version v23 (BM SPSS Statistics for Windows, Version 24.0. Armonk, NY: IBM Corp). Data collected include demographic characteristics, number and types of comorbidities, OTC products used, and number of prescription medications used. Descriptive statistics were expressed as mean and standard deviations for numerical data, and frequencies and percentages for categorical data. Differences in means between OTC users and non-users were assessed using the independent t test, while differences in proportions were tested with the Pearson’s Chi-square. Statistical significance was evaluated at the 0.05 significance level. The cohort in this study was also assessed for the use of pain medications [11].

## Results

A total of 404 older patients with a history of CVD were admitted to the cardiology service during the study period out of which 281 (69.6%) were taking at least one OTC product and were included in the study. Included patients had a mean age of 77.0 ± 7.5 years and 50.2% were males. Table 1 shows the characteristics of included patients. The most common comorbidities were hypertension (75.1%), followed by dyslipidemia (52.7%), coronary artery disease (CAD) (47.7%), and congestive heart failure (CHF) (45.9%). Table 2 shows the comorbidities with prevalence greater than 5% among OTC users. The average number of prescription products utilized by patients was 10.6 ± 3.7. Patients were taking a total of 659 OTC products; mean of 2.35 ± 1.57 and the range varied widely from 1 to 9 products. The most commonly used products were vitamins (37.3%), followed by laxatives (17%), minerals (13.6%), stomach acid reducers (9%), and analgesics (3.6%). The usage of specific single entity vitamin product was most commonly vitamin D, followed by vitamin B12, folic acid, and vitamin C. Table 3 describes the types of OTC products used. OTC users were found to be suffering from more comorbidities and received more prescription medications as compared to non-users (both *p*-values < 0.001) (Table 4). While gender and age did not have an impact on the use of OTC products, use of OTC products varied with history of certain medical conditions; patients with a history of atrial fibrillation (A Fib), sleep apnea, and gastro-esophageal reflux disease (GERD) were more likely to use OTC products (Table 5).

**Table 1:** Descriptive characteristics of OTC users

Number of OTC products	N	%
1	105	37.4
2	79	28.1
3	48	17.1
4	20	7.1
5	14	5
6	8	2.8
7	3	1.1
8	3	1.1
9	1	0.4
Mean (SD)	2.35	(1.57)
Median	2.00	
Age group		
65–69	56	19.9
70–74	58	20.6
75–79	56	19.9
80–84	60	21.4
85–89	32	11.4
90+	19	6.8
Mean (SD)	77.0	(7.5)
Median	76.0	
Gender		
Males	141	50.2
Females	140	49.8
Number of comorbidities		
1	1	0.4
2	2	0.7
3	16	5.7
4	52	18.5
5	39	13.9
6	37	13.2
7	42	14.9
8	40	14.2
9	29	10.3
10	9	3.2
11	4	1.4
12	4	1.4
13	1	0.4
14	3	1.1
15	1	0.4
16	1	0.4
Mean (SD)	6.5	(2.4)
Median	6.0

**Table 2:** Comorbidities with prevalence greater than 5% among OTC users

	N	%
Hypertension	211	75.1
Dyslipidemia	148	52.7
CAD	134	47.7
CHF	129	45.9
Diabetes mellitus	108	38.4
A Fib/flutter	106	37.7
CKD	89	31.7
COPD	61	21.7
Hypothyroidism	59	21.0
Cancer	55	19.6
Arthritis	54	19.2
GERD	54	19.2
Cardiac valve disease	46	16.4
BPH	45	16
CVA	42	14.9
PVD	36	12.8
Sleep apnea	34	12.1
Anemia	34	12.1
Gout	28	10
Depression	26	9.3
Pulmonary hypertension	24	8.5
Anxiety	23	8.2
GI bleed/ulcer	22	7.8
DVT /PE	22	7.8
Asthma	21	7.5
Obesity	21	7.5
Dementia	20	7.1
Neuropathy	19	6.8

*CAD*: coronary artery disease; *CHF*: congestive heart failure; *A Fib*: atrial fibrillation; *CKD*: chronic kidney disease; *COPD*: chronic obstructive pulmonary disease; *GERD*: gastro-esophageal reflux disease; *BPH*: benign prostatic hyperplasia; *CVA*: cerebrovascular accident; *PVD*: peripheral vascular disease; *GI*: gastrointestinal; *DVT*: deep venous thrombosis; *S*: pulmonary embolism

**Table 3:** Type of OTC products used by patients

Type of OTC Product	No (%)
Vitamins	**246 (37.3%)**
Vitamin D	94
Multi-vitamins	67
Vitamin B12	37
Folic acid	16
Vitamin C	8
Biotin	8
Vitamin B complex	7
Vitamin E	3
Vitamin B3 (niacin)	3
Vitamin B1	2
Vitamin B6	1
Laxatives	**112 (17%)**
Docusate	60
Polyethylene glycol (PEG)	34
Senna	8
Psyllium	6
Bisacodyl	4
Minerals	**90 (13.6%)**
Iron	33
Calcium	29
Magnesium	15
Potassium	11
Zinc	2
Stomach acid reducers	**59 (9%)**
Proton pump inhibitors (PPIs)	23
H2 antagonists	36
Analgesics	**24 (3.6%)**
Acetaminophen	17
Nonsteroidal anti-inflammatory drugs	7
Omega-3-acid	**19 (2.9%)**
Antihistamines	**18 (2.7%)**
Loratadine	11
Diphenhydramine	7
Coenzyme Q10	**11 (1.7%)**
Probiotics	**11 (1.7%)**
Other	**50 (7.6%)**
Fluticasone nasal spray	10
Guaifenesin	10
Melatonin	9
Nicotine	8
Glucosamine	4
Chondroitin	3
Cranberry	3
Artificial tears	3

**Table 4:** Characteristics of OTC users vs non users

	NON USERS OF OTC			USERS OF OTC			
	N	Mean	SD	N	Mean	SD	*p*-value
Age	123	75.7	7.1	281	77.0	7.5	0.097
No of prescription meds	123	8.4	3.7	281	10.6	3.7	< 0.001
No of comorbidities	123	5.2	2.1	281	6.5	2.4	< 0.001

**Table 5:** Mean of OTC products used by users characteristics

	N	Mean	SD	*p*-value
Age groups				
65–69	56	2.23	1.477	0.403
70–74	58	2.24	1.658	
75–79	56	2.39	1.67	
80–84	60	2.18	1.432	
85–89	32	2.66	1.45	
90+	19	2.84	1.803	
Gender				
Males	141	2.22	1.455	0.178
Females	140	2.47	1.664	
A Fib				
Absent	175	2.19	1.465	0.038
Present	106	2.59	1.695	
Sleep apnea				
Absent	247	2.25	1.504	0.006
Present	34	3.03	1.834	
GERD				
Absent	227	2.22	1.464	0.015
Present	54	2.89	1.85	

## Discussion

This study focused on examining the types of OTC products used by older adults with CVD and the association between use and several factors. According to our findings, OTC product use among older adults with CVD is quiet frequent. While the use of OTC and herbal products in patients with CVD across Canada was 67%, which is very similar to what we saw in our study (69.6%) [7], the methodological differences across studies, including heterogeneity in products studied, age, and the specific CVD prevent direct comparison between our study and other studies.

Vitamins were the most commonly used OTC products in our study which is similar to other studies conducted in Canada, Australia, and USA involving older adults or patients with CVD [7, 12–15]. The role of vitamins A, B, C, E, biotin, and β-carotene in CVD has been explored in several randomized controlled trials and results showed that they have no role in primary or secondary prevention of CVD and may even increase mortality in patients with pre-existing late-stage atherosclerosis [16–20]. Hence, the evidence is still insufficient to support the role of routine use of vitamins in patients with CVD. Furthermore, the FDA has issued a warning after an increase in the number of reported adverse effects, including one death following falsely low troponin results, caused by biotin use. Many lab tests that use biotin technology, including cardiovascular diagnostic tests and hormone tests, may generate incorrect results if there is biotin in patient’s specimen which may lead to inappropriate patient management or misdiagnosis. FDA advised patients to consult their clinicians if they are taking or thinking about taking biotin or any supplements containing biotin [21].

Vitamin D was the most commonly used vitamin in our study (38.2%). Identification of vitamin D receptors in the heart and vascular endothelial cells raised interest in the potential cardiovascular effects of vitamin D. Although several epidemiological studies have suggested that persons with low blood levels of vitamin D have increased risks of heart disease, stroke, and hypertension [22–25], meta-analyses and randomized controlled trials of vitamin D supplementation have failed to show cardiovascular benefits of this vitamin [26, 27].

Constipation is a common complaint in older adults and about one in six patients were on OTC laxatives in our study. Docusate was the most commonly used laxative despite the insufficient evidence to support its use in chronic constipation [28]. On the other hand, the second most commonly used laxative was polyethylene glycol (PEG), an agent with good quality evidence supporting its use in chronic constipation and is considered safe in long-term use [29, 30].

About one in ten patients used stomach acid reducers (H2 antagonists or proton pump inhibitors (PPIs)). According to the American Geriatrics Society (AGS), H2 antagonists’ doses have to be reduced due to varying levels of kidney function, a problem that is common in older adults while the PPI use beyond 8 weeks without justification should be avoided [31]. It is important that prescribers be aware of the use of stomach acid reducers in older adults and monitor their continued use to prevent the unnecessary cost, drug-drug interactions and side effects.

Non-steroidal anti-inflammatory drugs (NSAIDs) increase the risk of cardiovascular events as early as the first weeks of use, especially when used at higher doses. In addition, following NSAID use patients with heart disease or risk factors for it have a greater likelihood of heart attack or stroke than patients without these risk factors [32, 33]. In addition, the AGS recommends avoiding chronic NSAID use due to increased risk of gastrointestinal bleeding or peptic ulcer disease especially in patients on anticoagulants, or antiplatelet agents which is common in patients with CVD [31]. Patients should be counseled to take NSAIDs only if needed, at the lowest effective dose and for the shortest period of time.

A small portion of patients used herbal products. Unlike pharmaceutical products approved for the treatment of a specific disease, herbal products used by our patients have little published high quality evidence to support their use and are marketed without proof of efficacy or safety.

In our study, age and gender did not have an effect on the utilization of OTC products, which is consistent with findings from one study conducted across Canada involving adults with CVD [7]. Similarly, another study involving older adults living on the United States-Mexico border did not find any significant difference between men and women [34]. On the other hand, in a study conducted in South Australia, females as well as older adults between 65 and 79 years were more likely to use OTC products than those 80 years and older [12]. Our results suggest that some comorbidities were associated with an increased likelihood of using OTC products; A fib, sleep apnea, and GERD. Furthermore, OTC users tend to have more comorbidities and received more prescription medications as compared to non-users. These findings need to be confirmed by further studies.

Our study revealed that concurrent use of prescription and OTC products in older adults with CVD remains an important public health problem that needs to be monitored closely. Healthcare professionals need to be aware of the use of the OTC products in patients with CVD and encourage patients to report the use of such products. Furthermore, physicians and pharmacists should be aware of their potential for adverse reactions and drug interactions which is expected to be high among patients with CVD due to complex and multi drug regimens usually prescribed to this group of patients.

Our study is not without limitations. This is a retrospective chart review conducted at a single tertiary center in Alabama, USA, prevalence and types of OTC products may be different in more ethnically diverse populations. Patients may be unable to recall the OTC products they are taking due to poor health literacy, which can underestimate the prevalence of their use. In addition, incomplete documentation of the use of OTC products in patients’ chart cannot be excluded.

## Conclusion

In conclusion, the use of OTC products is common in older adults with CVD. Vitamins, laxatives, minerals, and stomach acid reducers were the most commonly used products. Clinicians treating patients with CVD should ask about OTC products usage and counsel patients about the risks and benefits associated with their use.

## Abbreviations

A Fib: Atrial fibrillation
AGS: American Geriatrics Society
CAD: Coronary artery disease
CHF: Congestive heart failure
CVD: Cardiovascular disease
GERD: Gastro-esophageal reflux disease
OTC: Over the counter
PEG: Polyethylene glycol
PPIs: Proton pump inhibitors

## References

[1] Benjamin EJ, Blaha MJ, Chiuve SE, Cushman M, Das SR, Deo R, de Ferranti SD, Floyd J, Fornage M, Gillespie C, Isasi CR (2017). Heart disease and stroke statistics—2017 update: a report from the American Heart Association. Circulation.

[2] de Souza Silva JE, Souza CA, da Silva TB, Gomes IA, de Carvalho Brito G, de Souza Araújo AA (2014). de Lyra-Júnior DP, da Silva WB, da Silva FA. Use of herbal medicines by elderly patients: a systematic review. Arch Gerontol Geriatr.

[3] Artz MB, Harnack LJ, Duval SJ, Armstrong C, Arnett DK, Luepker RV (2006). Use of nonprescription medications for perceived cardiovascular health. Am J Prev Med.

[4] Açıkgöz SK, Açıkgöz E, Topal S, Okuyan H, Yaman B, Er O, Şensoy B, Balcı MM, Aydoğdu S (2014). Effect of herbal medicine use on medication adherence of cardiology patients. Complement Ther Med..

[5] Krasuski RA, Michaelis K, Eckart RE (2006). The cardiovascular patient's perceptions of complementary and alternative medicine. Clin Cardiol.

[6] Albert NM, Rathman L, Ross D, Walker D, Bena J, McIntyre S, Philip D, Siedlecki S, Lovelace R, Fogarty AM, Maikut B (2009). Predictors of over-the-counter drug and herbal therapies use in elderly patients with heart failure. J Card Fail.

[7] Pharand C, Ackman ML, Jackevicius CA, Paradiso-Hardy FL, Pearson GJ (2003). Use of OTC and herbal products in patients with cardiovascular disease. Ann Pharmacother.

[8] Dal Corso E, Bondiani AL, Zanolla L, Vassanelli C (2007). Nurse educational activity on non-prescription therapies in patients with chronic heart failure. Eur J Cardiovasc Nurs.

[9] Gardiner P, Graham RE, Legedza AT, Eisenberg DM, Phillips RS (2006). Factors associated with dietary supplement use among prescription medication users. Arch Intern Med.

[10] Hensrud DD, Engle DD, Scheitel SM (1999). Underreporting the use of dietary supplements and nonprescription medications among patients undergoing a periodic health examination. Mayo Clin Proc..

[11] Kabbara WK, Dimassi H, Sheikh-Taha M (2018). Patterns of pain medication use in older individuals with cardiovascular disease. Curr Med Res Opin.

[12] Goh LY, Vitry AI, Semple SJ, Esterman A, Luszcz MA (2009). Self-medication with over-the-counter drugs and complementary medications in South Australia's elderly population. BMC Complement Altern Med.

[13] Stys T, Stys A, Kelly P, Lawson W (2004). Trends in use of herbal and nutritional supplements in cardiovascular patients. Clin Cardiol.

[14] Qato DM, Alexander GC, Conti RM, Johnson M, Schumm P, Lindau ST (2008). Use of prescription and over-the-counter medications and dietary supplements among older adults in the United States. JAMA.

[15] Alherbish A, Charrois TL, Ackman ML, Tsuyuki RT, Ezekowitz JA (2011). The prevalence of natural health product use in patients with acute cardiovascular disease. PLoS One.

[16] Honarbakhsh S, Schachter M (2008). Vitamins and cardiovascular disease. Br J Nutr.

[17] Albert CM, Cook NR, Gaziano JM, Zaharris E, MacFadyen J, Danielson E, Buring JE, Manson JE (2008). Effect of folic acid and B vitamins on risk of cardiovascular events and total mortality among women at high risk for cardiovascular disease: a randomized trial. JAMA.

[18] Sesso HD, Buring JE, Christen WG, Kurth T, Belanger C, MacFadyen J, Bubes V, Manson JE, Glynn RJ, Gaziano JM (2008). Vitamins E and C in the prevention of cardiovascular disease in men: the Physicians' health study II randomized controlled trial. JAMA.

[19] Vivekananthan DP, Penn MS, Sapp SK, Hsu A, Topol EJ (2003). Use of antioxidant vitamins for the prevention of cardiovascular disease: meta-analysis of randomised trials. Lancet.

[20] Omenn GS, Goodman GE, Thornquist MD, Balmes J, Cullen MR, Glass A, Keogh JP, Meyskens FL, Valanis B, Williams JH, Barnhart S (1996). Effects of a combination of beta carotene and vitamin a on lung cancer and cardiovascular disease. N Engl J Med.

[21] FDA (2017, 11) Biotin (Vitamin B7): Safety Communication - May Interfere with Lab Tests. Retrieved from: https://www.fda.gov/medicaldevices/safety/alertsandnotices/ucm586505.htm. Accessed 24 Nov 2018.

[22] Giovannucci E, Liu Y, Hollis BW, Rimm EB (2008). 25-hydroxyvitamin D and risk of myocardial infarction in men: a prospective study. Arch Intern Med.

[23] Wang TJ, Pencina MJ, Booth SL, Jacques PF, Ingelsson E, Lanier K, Benjamin EJ, D’Agostino RB, Wolf M, Vasan RS (2008). Vitamin D deficiency and risk of cardiovascular disease. Circulation.

[24] Dobnig H, Pilz S, Scharnagl H, Renner W, Seelhorst U, Wellnitz B, Kinkeldei J, Boehm BO, Weihrauch G, Maerz W (2008). Independent association of low serum 25-hydroxyvitamin d and 1,25-dihydroxyvitamin d levels with all-cause and cardiovascular mortality. Arch Intern Med.

[25] Anderson JL, May HT, Horne BD (2010). Relation of vitamin D deficiency to cardiovascular risk factors, disease status, and incident events in a general healthcare population. Am J Cardiol.

[26] Pilz S, Verheyen N, Grübler MR, Tomaschitz A, März W, Vitamin D (2016). cardiovascular disease prevention. Nat Rev Cardiol.

[27] Al Mheid I, Quyyumi AA (2017). Vitamin D and cardiovascular disease: controversy unresolved. J Am Coll Cardiol.

[28] Singh S, Rao SS (2010). Pharmacologic management of chronic constipation. Gastroenterol Clin N Am.

[29] DiPalma JA, vB Cleveland M, McGowan J, Herrera JL (2007). A randomized, multicenter, placebo-controlled trial of polyethylene glycol laxative for chronic treatment of chronic constipation. Am J Gastroenterol.

[30] Corazziari E, Badiali D, Bazzocchi G, Bassotti G, Roselli P, Mastropaolo G, Lucà MG, Galeazzi R, Peruzzi E (2000). Long term efficacy, safety, and tolerabilitity of low daily doses of isosmotic polyethylene glycol electrolyte balanced solution (PMF-100) in the treatment of functional chronic constipation. Gut.

[31] By the American Geriatrics Society 2015 Beers Criteria Update Expert Panel (2015). American Geriatrics Society 2015 Beers criteria update expert panel. American Geriatrics Society 2015 updated beers criteria for potentially inappropriate medication use in older adults. J Am Geriatr Soc..

[32] Bueno H, Bardají A, Patrignani P, Martín-Merino E, García-Rodríguez LA (2010). Use of non steroidal anti-inflammatory drugs and type specific risk of acute coronary syndrome. Am J Cardiol.

[33] Fanelli A, Ghisi D, Aprile PL, Lapi F (2017). Cardiovascular and cerebrovascular risk with nonsteroidal anti-inflammatory drugs and cyclooxygenase 2 inhibitors: latest evidence and clinical implications. Ther Adv Drug Saf..

[34] Loya AM, González-Stuart A, Rivera JO (2009). Prevalence of polypharmacy, polyherbacy, nutritional supplement use and potential product interactions among older adults living on the United States-Mexico border. Drugs & Aging.

